# The Role of Health, Social Network, and Race in Advance Care Planning Among Medicare Beneficiaries

**DOI:** 10.1093/geroni/igaa057.227

**Published:** 2020-12-16

**Authors:** Jung Kwak, Heehyul Moon, Soonhee Roh

**Affiliations:** 1 University of Texas at Austin, Austin, Texas, United States; 2 University of Louisville, Louisville, Kentucky, United States; 3 University of South Dakota, Sioux Falls, South Dakota, United States

## Abstract

Advance care planning (ACP) is linked with high-quality end-of-life outcomes. However, ACP engagement level among older adults varies significantly by demographic, social, and health characteristics. In this study, we sought to identify characteristics associated with informal and formal ACP, in order to inform development of targeted education and outreach efforts that are tailored to diverse groups of older adults. The data came from a nationally representative study of Medicare beneficiaries living in communities, the National Health and Aging Trends Study (Round 8, N= 5,547). Multivariable logistic regressions were conducted to identify individual characteristics (i.e., race/ethnicity, age, gender, income, functional disability, cognitive function, perceived health, and numbers of people in social networks) associated with ACP engagement. Rates of informal ACP (talking to someone), and formal ACP, completing a healthcare power of attorney (HPOA) and a living will (LW), were 56%, 60.5%, and 56% accordingly. Logistic regression showed that individuals who were married or had a larger social network, and had higher functional impairment and health needs were significantly more likely to engage in both informal and formal ACP. However, individuals with memory problems (only informal ACP) and African Americans and Hispanics were significantly less likely to engage in both informal and formal ACP. African Americans without dementia were more likely to have completed HPOA compared with Whites. Findings suggest an important role of social network, and functional and cognitive health in ACP with implications for developing targeted outreach efforts in faith-based or social group settings, and healthcare settings.

